# Investigating the survival mechanisms of *Lactiplantibacillus plantarum* QZW5 under repeated freeze–thaw stress: an integrated study using biochemical assays, environmental cryo-electron microscopy, and multi-omics approaches

**DOI:** 10.3389/fmicb.2026.1873698

**Published:** 2026-09-16

**Authors:** Miao Zhang, Shuang Wen, Zelin Zhang, Emmanuel Tenakwa, Naana Akufo, Huili Pang, Lei Wang, Guofang Wu, Terry Ansah, Dongsheng Lei, Yimin Cai, Zhongfang Tan

**Affiliations:** 1 Zhengzhou Research Base, National Key Laboratory of Cotton Bio-breeding and Integrated Utilization, School of Agriculture and Biomanufacturing, Zhengzhou University, Zhengzhou, China; 2 Henan Key Laboratory of Ion Beam Green Agriculture Bioengineering, School of Agriculture and Biomanufacturing, Zhengzhou University, Zhengzhou, Henan, China; 3 Key Laboratory of Magnetism and Magnetic Functional Materials, School of Physical Science and Technology, Electron Microscopy Centre of Lanzhou University, Lanzhou University, Lanzhou, China; 4 Plateau Livestock Genetic Resources Protection and Innovative Utilization Key Laboratory of Qinghai Province, Key Laboratory of Animal Genetics and Breeding onTibetan Plateau, Ministry of Agriculture and Rural Affairs, Academy of Animal Science and Veterinary Medicine, Qinghai University, Xining, Qinghai, China; 5 Faculty of Agriculture, Food and Consumer Sciences, University for Development Studies, Tamale, Ghana; 6 State Key Laboratory for Animal Disease Control and Prevention, College of Veterinary Medicine, Lanzhou University, Lanzhou Veterinary Research Institute, Chinese Academy of Agricultural Sciences, Lanzhou, China

**Keywords:** freeze–thaw tolerance, gene expression regulation, *Lactiplantibacillus plantarum* QZW5, metabolic reprogramming, multi-omics analysis

## Abstract

**Introduction:**

Repeated freeze–thaw cycles represent a critical stress factor in frozen food processing, leading to a significant decline in the survival and fermentation performance of lactic acid bacteria.

**Methods:**

This study investigated the tolerance mechanism of *Lactiplantibacillus plantarum* QZW5, a resistant strain, after 10 cycles of alternating freezing (−20 °C) and thawing (20 °C). An integrated approach combining physiological and biochemical assays, environmental cryo-electron microscopy, multi-omics analysis, and qPCR validation was employed.

**Results and Discussion:**

The results showed after freeze–thaw treatment, QZW5 retained an 85.20%survival rate with a 14.80% reduction in growth rate, accompanied by gradual fermentation pH decline. Its antibacterial activity decreased markedly; for example, the inhibition zone against *Micrococcus luteus* decreased from 20.36 ± 1.77 mm to 10.64 ± 0.10 mm (≈48% reduction),and ampicillin susceptibility increased by around 18% (inhibition zone from 24.76 ± 0.35 mm to 29.11 ± 0.17 mm),while its biofilm-forming capacity remained stable with OD₅₇₀ values of 1.90 ± 0.23 (CK) and 1.98 ± 0.19 (FT, *p* > 0.05). Morphological alterations included serrated cell edges and intracellular granule deposition. At the molecular level, QZW5 activated a coordinated stress response: the upregulation of genes related to glyoxylate metabolism, ABC transporters, purine/pyrimidine metabolism, and phenazine synthesis enhanced nutrient uptake and protective barrier formation; meanwhile, the downregulation of genes involved in oxidative phosphorylation and peptidoglycan synthesis reduced energy expenditure and was accompanied by programmed cell deathlike responses. Quantitative PCR (qPCR) validation confirmed that core genes such as fabZ and purN were sharply downregulated (relative expression dropped from ~2070 and ~589 to below 65 and 27 respectively), verifying that QZW5 adapts to stress by “inhibiting energy consumption while enhancing repair. In conclusion, QZW5 achieves freeze–thaw tolerance through the synergistic interplay of morphological adaptation, metabolic reprogramming, and gene regulation. This study provides theoretical and technical support for the development and application of QZW5 as a starter culture for frozen foods.

## 1 Introduction

Lactic Acid Bacteria (LAB) are key microorganisms in the food industry, valued for their ability to enhance nutritional value, improve flavor, and extend the shelf life of products through natural fermentation processes (Chen et al., 2025; Ibrahim et al., 2023). Their applications have significantly expanded into the frozen food sector, serving not only as fermentation starters but also as functional ingredients contributing to texture modification, biopreservation, and probiotic delivery (Brillet et al., 2005). This broad utility underscores the importance of their viability and functionality in frozen matrices.

However, the repeated freeze–thaw cycles inherent to many food processing and storage protocols inflict severe physiological stress on LAB. The formation of intracellular ice crystals, membrane phase transition (Chen et al., 2020), protein denaturation, and osmotic imbalance collectively lead to a dramatic decline in cell survival and functional efficacy (Li et al., 2025). This damage constitutes a major bottleneck for the wider application of LAB in frozen food systems.

Current strategies to mitigate freeze–thaw damage predominantly rely on external interventions, such as the addition of cryoprotectants (e.g., sugars, peptides) (Zhu et al., 2025) or process optimization (Wang et al., 2022). While effective, these approaches often do not address the intrinsic tolerance mechanisms of the bacterial cells themselves. Consequently, there is a compelling need to elucidate the fundamental molecular and cellular adaptations that enable specific LAB strains to withstand such stress, particularly those isolated from naturally challenging environments.

Our previous work isolated *Lactiplantibacillus plantarum* QZW5 from a high-altitude region. This strain demonstrates remarkable resilience, maintaining high viability and altered functional properties (e.g., modulated antibiotic resistance) after repeated freeze–thaw cycles (Li et al., 2023; Liu et al., 2024). These observations suggest that QZW5 possesses robust, intrinsic adaptive mechanisms, the details of which remain unexplored.

To bridge this knowledge gap, the present study employed an integrated multi-modal approach. We combined physiological profiling, environmental cryo-electron microscopy for ultrastructural analysis, alongside transcriptomic and metabolomic investigations. This comprehensive strategy aimed to systematically decode the survival mechanisms of *L. plantarum* QZW5 under repeated freeze–thaw stress, from phenotypic changes to underlying gene expression and metabolic reprogramming. The findings are expected to provide a solid theoretical foundation for the targeted application of this robust strain and inform future strategies for developing freeze–thaw-tolerant LAB cultures.

## 2 Materials and methods

### 2.1 Bacterial strain and freeze–thaw treatment

Lactiplantibacillus plantarum QZW5 was isolated from naturally fermented traditional dairy products sampled from the Qinghai-Tibet Plateau, China. The preserved strain was deposited in the China General Microbiological Culture Collection Center (CGMCC). The stock culture was stored at −80 °C in MRS medium containing 20% (v/v) sterile glycerol, and the strain was activated twice consecutively in fresh MRS broth at 30 °C prior to all experimental treatments.

The *Lactiplantibacillus plantarum* QZW5 strain was activated on MRS agar under anaerobic conditions at 30 °C. Activated colonies were inoculated into MRS broth, and both the freeze–thaw (FT) treatment group and the contronl (CK) group were acclimated at 5 °C for 7 days to unify their physiological baseline. After acclimation, the FT group was subjected to 10 repeated freeze–thaw (FT) cycles: freezing at −20 °C for 24 h followed by thawing at 20 °C. The CK group was transferred to 30 °C for continuous incubation, six independent biological replicates were set for each group.

### 2.2 Survival rate and pH measurement

The pH of bacterial cultures was measured directly using a pH meter. For survival rate determination, cultures were serially diluted, plated on MRS agar, and incubated anaerobically at 30 °C for 48 h before colony counting.

Survival rate calculation formula:


$$\text{Survival rate}=\frac{\text{Colony count after freeze}-\text{thaw}}{\text{Colony count before freeze}-\text{thaw}}\times 100\%$$

All measurements were performed in triplicate.

### 2.3 Cryo-electron microscopy (Cryo-EM)

Mid-logarithmic-phase bacterial pellets from control (CK) and freeze–thaw (FT) groups were collected and diluted to OD_600_ ≈ 0.6 with pre-cooled sterile PBS (pH 6.8). For cryo-EM specimen preparation, 3 μL diluted bacterial suspension was loaded onto glow-discharged Quantifoil R2/2 carbon grids. Excess liquid was blotted for 2.5 s under 100% humidity at 4 °C, and grids were instantly plunge-frozen in liquid ethane cooled by liquid nitrogen using Vitrobot Mark IV (Thermo Fisher Scientific, USA). Prepared frozen grids were transferred into a Talos Arctica cryo-transmission electron microscope (Thermo Fisher, USA) operated at an acceleration voltage of 200 kV. Micrographs were captured at 39,000 × magnification with defocus values ranging from −1.0 μm to −2.5 μm. Raw microscopic images were processed, measured and statistically analyzed using ImageJ v1.54f and Relion 3.1 software.

### 2.4 Biofilm assay

Biofilm formation was quantified using a crystal violet staining method. Bacteria were cultured statically in glass tubes for 48 h. After washing, adherent biofilms were stained with 0.1% crystal violet, dissolved in 33% acetic acid, and the absorbance was measured at 570 nm. Uninoculated medium served as the blank control. Six biological replicates were analyzed.

### 2.5 Antibacterial activity

Antibacterial activity was assessed against a panel of standard pathogenic bacteria using the agar well diffusion method. Pathogen-inoculated soft agar was overlaid on nutrient agar. For cell-free supernatant preparation, bacterial pellets were collected by centrifugation at 8000 × g for 10 min at 4 °C after terminal cultivation. The collected supernatant was filtered through a 0.22 μm sterile microporous filter to remove residual intact bacterial cells, and the filtrate was defined as CFS for subsequent activity assays.

### 2.6 Antibiotic susceptibility testing

Susceptibility was determined by the standard disk diffusion method on MRS agar, using commercial antibiotic disks (Oxoid). Zones of inhibition were measured after 24 h at 37 °C and interpreted according to CLSI guidelines (Supplementary Table 1). Tests were performed in triplicate.

### 2.7 Proteomic and metabolomic analysis

For proteomics detection, total cellular proteins were extracted from cell pellets using urea-based lysis buffer supplemented with complete protease inhibitor cocktail on ice. Total protein concentration was quantified via BCA colorimetric assay before subsequent processing. Equal-quantity protein samples were denatured and reduced by dithiothreitol (DTT), followed by cysteine alkylation with iodoacetamide (IAA) under dark conditions. Trypsin was added at a trypsin/protein mass ratio of 1:50 for overnight enzymatic digestion at 37 °C. Desalted peptide mixtures were separated on an EASY-nLC 1,200 ultra-high-performance liquid chromatography system, and tandem mass spectrometry was performed on an Orbitrap Exploris 480 mass spectrometer (Thermo Fisher Scientific, USA). Raw proteomic MS data were annotated and quantified against the UniProt Lactiplantibacillus plantarum reference database with MaxQuant v1.6.17 software.

Untargeted metabolomic profiling was entrusted to Majorbio Bio-pharm Technology Co. Ltd. (Shanghai, China). Intracellular metabolites were extracted from lyophilized bacterial powder using methanol - acetonitrile - aqueous mixed extractant. Chromatographic separation and mass spectrometry acquisition were carried out on the consistent nLC-MS/MS platform as proteomic analysis. All acquired metabolome data were annotated against HMDB, METLIN and KEGG databases via Majorbio Cloud online platform for differential metabolite screening and pathway enrichment analysis.

### 2.8 Transcriptomic analysis

Total RNA was extracted from samples using a commercial kit. RNA quality was assessed, and sequencing libraries were prepared. Paired-end sequencing was performed on an Illumina platform. After quality control, reads were aligned to the reference genome. Differential gene expression analysis was conducted using DESeq2, with pathway enrichment performed via KEGG. A significance threshold of *p* < 0.05 (FDR-adjusted) was applied.

### 2.9 Quantitative real-time PCR (RT-qPCR)

Selected differentially expressed genes were validated by RT-qPCR. RNA was reverse transcribed to cDNA, which was then amplified using gene-specific primers on a real-time PCR system. The 16S rRNA gene, a stable housekeeping gene for Lactiplantibacillus plantarum, served as the internal reference, and relative expression levels were calculated using the 2^(-ΔΔCt) method.

### 2.10 Statistical analysis

All statistical analyses were performed via GraphPad Prism 9.0 and SPSS 26.0. Shapiro–Wilk test and Levene’s test were conducted to verify normality and homoscedasticity before parametric tests. Two-group comparisons adopted independent t-test, and multiple-group comparisons used one-way ANOVA with Tukey’s post-hoc test.

All experiments including phenotypic and molecular assays contained six biological replicates (*N* = 6). The technical triplicates mentioned only represent repeated measurements on each biological sample to reduce detection deviation; all final data were calculated from six independent biological samples without pooled single samples. Statistical significance was set at *p* < 0.05.

## 3 Results and discussion

### 3.1 Changes in biochemical characteristics of QZW5 before and after freeze–thaw cycles

Table 1 summarizes the physiological and biochemical changes observed in *Lactiplantibacillus plantarum* QZW5 after undergoing 10 alternating freeze–thaw cycles, alternating between −20 °C and 20 °C. Compared with the control group (100.00%), the survival rate of QZW5 decreased to 85.20% after freeze–thaw treatment, indicating partial cell damage without substantial death. This high survival rate reflects QZW5’s intrinsic freeze–thaw tolerance, supporting its potential as a stable starter culture for use in frozen food processing.

**Table 1:** Properties of QZW5 before and after freeze–thaw.

Indicators	QZW5 (CK)	QZW5 (FT)
Survival rate	100.00%	85.20%
Biofilm (OD value)	1.90 ± 0.23^a^	1.98 ± 0.19^a^
Antimicrobial activity presented as inhibition zone (mm)		
*Micrococcus luteus* ATCC 4698	20.36 ± 1.77^a^	10.64 ± 0.10^b^
*Bacillus subtilis* ATCC6633	13.18 ± 0.29^a^	8.61 ± 0.16^b^
*Escherichia coli* ATCC11775	11.18 ± 2.02^a^	8.51 ± 0.22^b^
*Listeria monocytogenes* ATCC 19115	19.60 ± 2.51^a^	11.23 ± 2.02^b^
*Staphylococcus aureus* ATCC29213	21.28 ± 1.48^a^	10.32 ± 0.60^b^
*Salmonella enterica* ATCC 43971	13.60 ± 0.22^a^	11.01 ± 0.89^b^
*Pseudomonas aeruginosa* ATCC27853	16.67 ± 0.91^a^	9.32 ± 0.10^b^
Antibiotic resistance presented as inhibition zone (mm)		
Ampicillin	24.76 ± 0.35^a^	29.11 ± 0.17^b^
Erythromycin	20.79 ± 0.33^a^	23.07 ± 0.89^b^
Colistin sulphate	-	-
Penisillin G	17.72 ± 0.54^a^	19.27 ± 0.59^b^
Cioprofloxacin	-	-
Gentamicin	7.91 ± 0.11^a^	9.06 ± 0.16^b^
CHL Oramphenicol	25.21 ± 0.78^a^	26.82 ± 0.32^b^
Vancomycin	-	-
Rifampicin	16.70 ± 0.25^a^	18.72 ± 0.57^b^
TETracycline	17.94 ± 0.34^a^	23.00 ± 0.08^b^

^a,b^Values in the same raw of same item with different superscripts differ significantly(*p* < 0.05).

Supplementary Figure 1 depicts the dynamic pH changes of *Lactiplantibacillus plantarum* QZW5 over a period of 0–84 h under normal culture at 28 °C, as well as following 10 repeated freeze–thaw cycles. The 10 repeated freeze–thaw cycles significantly impaired the early acid-producing capacity of QZW5, leading to a notable decrease in pH reduction during fermentation. However, the difference compared to the control group was not significant after 84 h.

The biofilm-forming capacity remained stable, with OD₅₇₀ values of the CK group at 1.90 ± 0.23 and the FT group at 1.98 ± 0.19, showing no significant difference (*p* > 0.05, labeled “a” in Table 1). Stable biofilms, composed of extracellular polysaccharides, proteins, and nucleic acids, likely helped to reduce ice crystal damage and maintain water balance, serving as a key factor in QZW5’s resistance to freeze–thaw conditions.

In addition, the FT group exhibited significantly smaller inhibition zones against 7 indicator strains (e.g., Micrococcus sp., *Escherichia coli*) compared to the CK (*p* < 0.05, labeled “b” in Table 1), with the inhibition zone against Micrococcus sp. shrinking by ~48% (from 20.36 ± 1.77 mm to 10.64 ± 0.10 mm). This reduction in antibacterial activity may be due to impaired synthesis or secretion of antimicrobial substances (such as bacteriocins) which could result from damage to membrane transporters or ribosomal dysfunction caused by freeze–thaw cycles. For bio-preservative applications in frozen foods, optimizing the dosage of QZW5’s or combining it with other preservatives is recommended to compensate for its reduced efficiency.

In contrast, the FT group showed reduced antibiotic resistance, as reflected by an ~18% increase in the ampicillin inhibition zone (from 24.76 ± 0.35 mm to 29.11 ± 0.17 mm), with a similar trend observed for erythromycin (*p* < 0.05). This decreased resistance may be linked to stress-induced upregulation of resistance-related genes, such as efflux pumps. However, the potential risk of horizontal transfer of resistance genes to pathogens in food matrices requires further biosafety evaluation.

### 3.2 Cryo-Electron microscopy observations before and after freeze–thaw cycles

Environmental cryo-electron microscopy revealed that the causal linkage between repeated freeze–thaw stress and progressive ultrastructural damage of *Lactiplantibacillus plantarum* QZW5 cells (Figure 1). Under normal culture conditions without freeze–thaw treatment, CK-group cells displayed regular intact rod morphology with smooth, well-defined cell wall and membrane boundaries, uniform cytoplasmic density and homogeneous intracellular structure (Figures 1a–p). Following 10 successive freeze–thaw cycles, most cells retained their gross rod contour yet suffered severe subcellular structural deterioration (Figures 1a–p).

**Figure 1:**
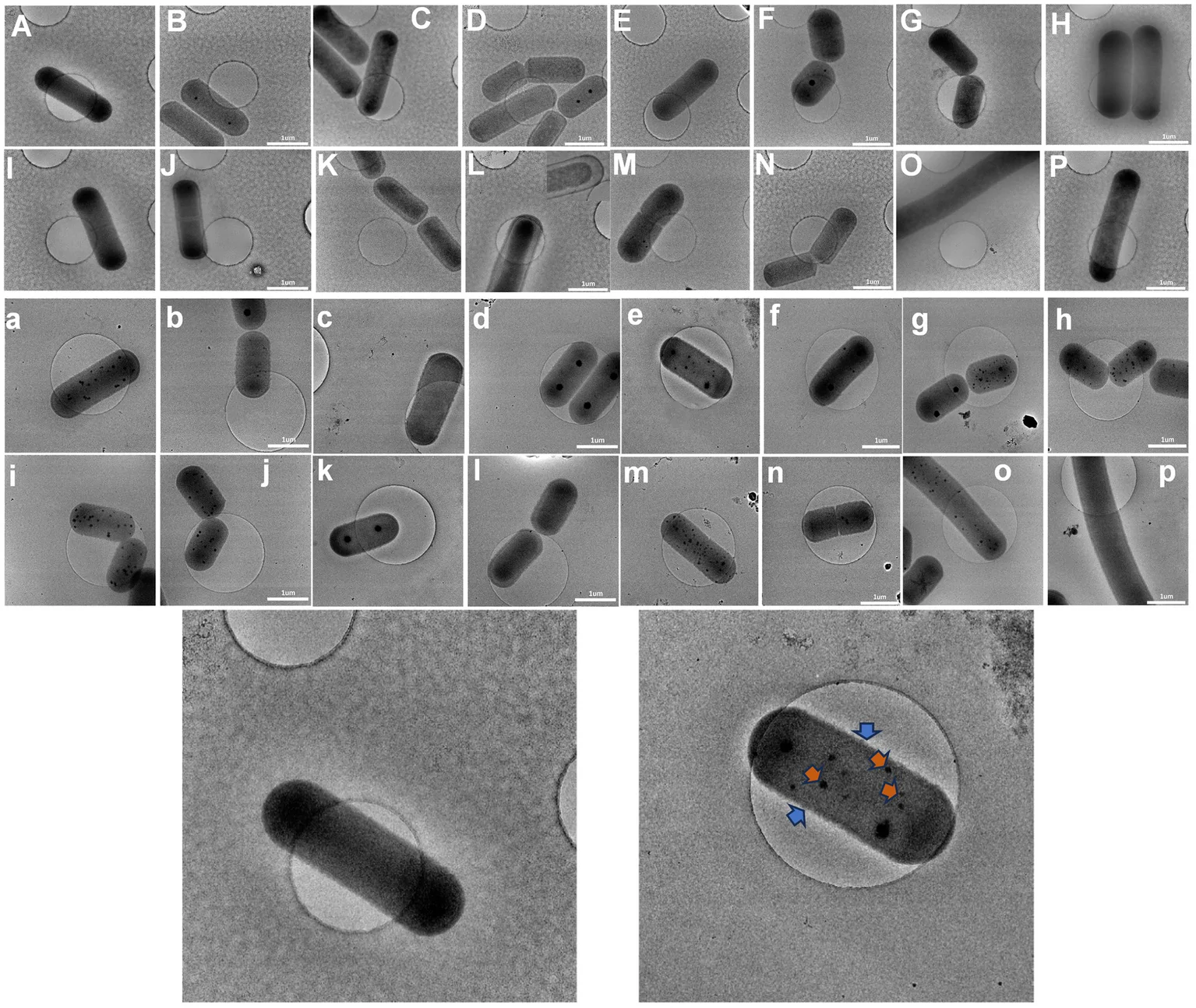
Cryo-electron microscopic morphological observation of *Lactiplantibacillus plantarum* QZW5 before and after freeze–thaw. (1.1) Morphological observation of cells before freeze–thaw **(a–p)**; morphological observation of cells after freeze–thaw **(a–p)**. (1.2) I Cell morphology before freeze–thaw; II Cell morphology after freeze-thaw.

High-magnification observation (Figure 1) further detailed two core injury phenotypes induced by freeze–thaw-derived ice crystal piercing and periodic osmotic fluctuation: irregular serrated cell peripheries (blue arrows) originating from partial rupture and shrinkage of peptidoglycan cell wall and cytoplasmic membrane, which triggered mild leakage of intracellular soluble metabolites; abundant electron-dense black granular inclusions scattered inside cytoplasm (orange arrows), which were ascribed to irreversible denaturation and aggregation of intracellular proteins as well as accumulated precipitated purine/pyrimidine metabolic intermediates. Such structural lesions were functionally consistent with our multi-omics data: the remarkable upregulation of purine and pyrimidine metabolism pathways exactly reflected cellular compensation for leaked nucleotide precursors and accumulated cytoplasmic precipitates after membrane damage, while perturbed peptidoglycan synthesis corresponded to compromised cell wall integrity.

Notably, QZW5 still achieved a final survival rate of 85.20% after repeated freeze–thaw damage. This survival outcome verifies that the strain launches a coordinated adaptive coping system to counteract structural injuries, including inducible membrane-repairing proteins and compatible solute accumulation, which collectively offset freeze–thaw-derived physical damage and sustain cellular physiological viability.

### 3.3 Transcriptome analysis results

#### 3.3.1 Transcriptomic response and pathway enrichment analysis of QZW5 under freeze–thaw stress

The X-axis represents the names of pathways; the Y-axis represents the rich factor [defined as the ratio of the number of genes annotated to the pathway in the gene set (Sample number) to the number of genes annotated to the pathway in all genes (Background number). A larger rich factor indicates a higher degree of enrichment]. The color indicates the significance of enrichment, i.e., *p*-value. Darker colors indicate more significant enrichment of the pathway, where *p*-value < 0.001 is marked as ***, *p*-value < 0.01 as **, and *p*-value < 0.05 as *. The color gradient on the right indicates the magnitude of *p*-values.

Transcriptome analysis revealed systematic gene expression reprogramming in QZW5 under repeated freeze–thaw (FT) cycles. Principal component analysis showed a clear separation between the control (CK) and FT groups, confirming the profound impact of the stress (Figure 2a). A large number of differentially expressed genes (DEGs) were identified (Figure 2b), and KEGG enrichment analysis indicated that the transcriptional response was strategically focused on core survival mechanisms.

**Figure 2:**
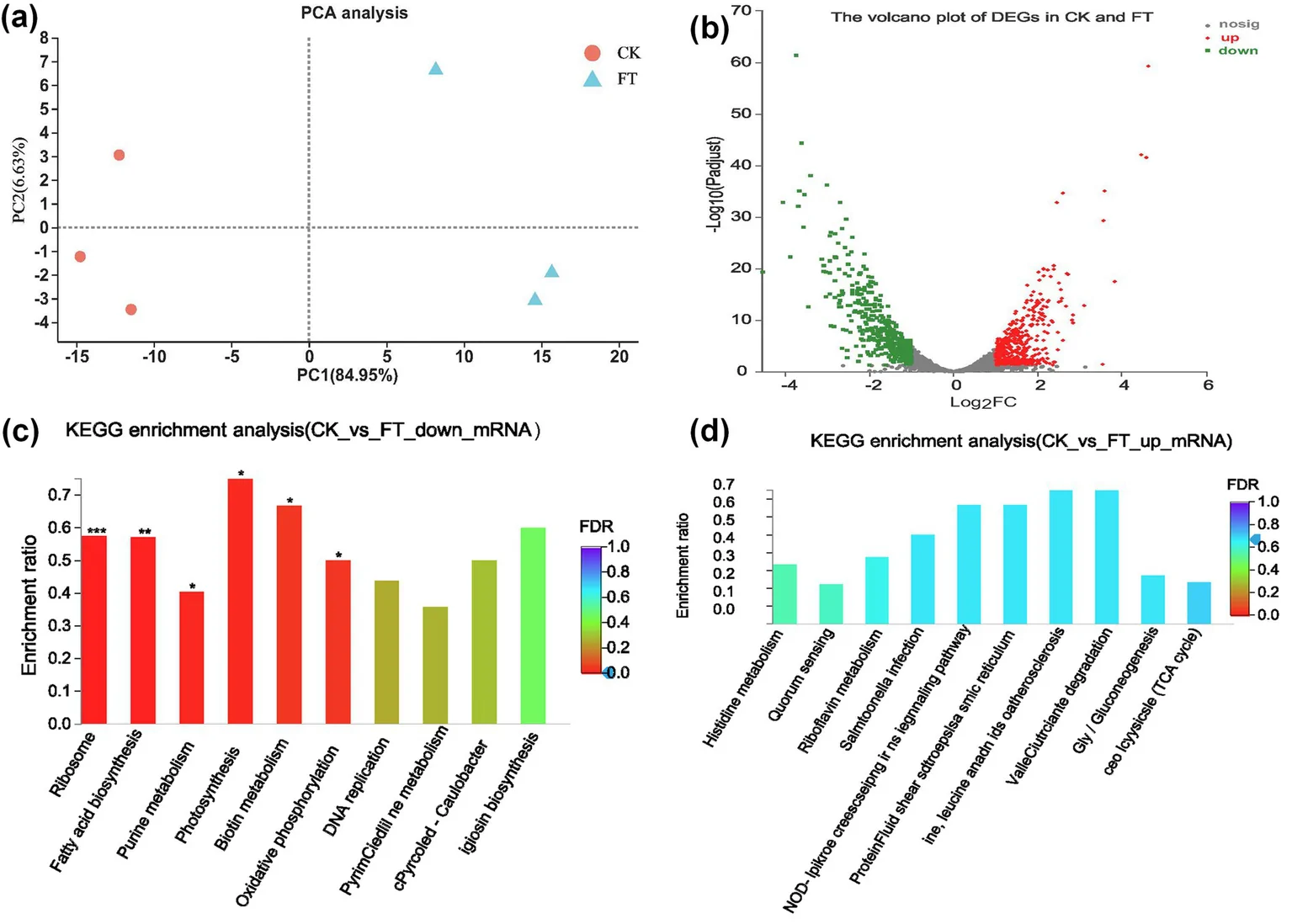
PCA analysis **(a)**, volcano plot of DEGs in CK and FT **(b)**, and KEGG pathway enrichment of down- **(c)** and up-regulated **(d)** DEGs in CK and FT.

The distinct separation between CK and FT groups in PCA demonstrates that repeated freeze–thaw cycles induce a global gene expression reprogramming in QZW5, a typical adaptive response to extreme environments (Garg et al., 2025). KEGG enrichment analysis revealed that the downregulated DEGs were significantly enriched in ribosome biogenesis, fatty acid biosynthesis, and biotin metabolism pathways (Figure 2c). In contrast, the upregulated DEGs were enriched in nitrogen metabolism and glutathione metabolism pathways (Figure 2d). This pattern indicates a coordinated shift from growth-oriented processes to stress defense and homeostasis maintenance.

The downregulation of ribosome-related genes suggests an “energy-saving strategy” under stress by reducing energy-intensive protein synthesis, potentially redirecting resources to critical repair processes (Peng et al., 2024). Concurrently, the downregulation of fatty acid biosynthesis genes may relate to adaptive adjustments in membrane lipid composition, such as increasing unsaturated fatty acids to maintain membrane fluidity at low temperatures (Geng et al., 2025). This aligns with cryo-EM observations of maintained membrane integrity despite damage.

The upregulation of glutathione metabolism genes directly enhances antioxidant capacity to scavenge reactive oxygen species generated during freeze–thaw cycles, mitigating oxidative damage (He et al., 2025). The upregulation of nitrogen metabolism genes likely helps rebalance intracellular nitrogen utilization and amino acid supply, supporting the synthesis of stress-related proteins (Riaz et al., 2025). Furthermore, differential expression of multiple F₀F₁ ATP synthase subunits (Supplementary Table 10) indicates a fine-tuned regulation of energy production to meet the altered demands under stress (Almeida-Oliveira et al., 2023).

Analysis of the 20 genes uniquely expressed in the FT group provided further insight into specialized adaptive mechanisms (Figure 3a). KEGG enrichment of these FT-specific genes highlighted pathways for biosynthesis of aromatic amino acids (phenylalanine, tyrosine, tryptophan), phenazine biosynthesis, and biofilm formation (Figure 3b). This enrichment suggests supplementary adaptation strategies: aromatic amino acids supply precursors for stress proteins and signaling molecules; phenazines may offer additional antioxidant and antimicrobial protection (Wu Y. et al., 2025); and genes related to biofilm formation (e.g., trpG) corroborate the observed biofilm stability, which can serve as a physical barrier. Key FT-specific genes included transporters for ion and carbohydrate homeostasis (e.g., DASS and PTS families), metabolic enzymes like ttdA involved in glyoxylate metabolism (Proffitt et al., 2022), and transcriptional regulators (MarR, TetR/AcrR families) for global response coordination (Wu H. et al., 2025).

**Figure 3:**
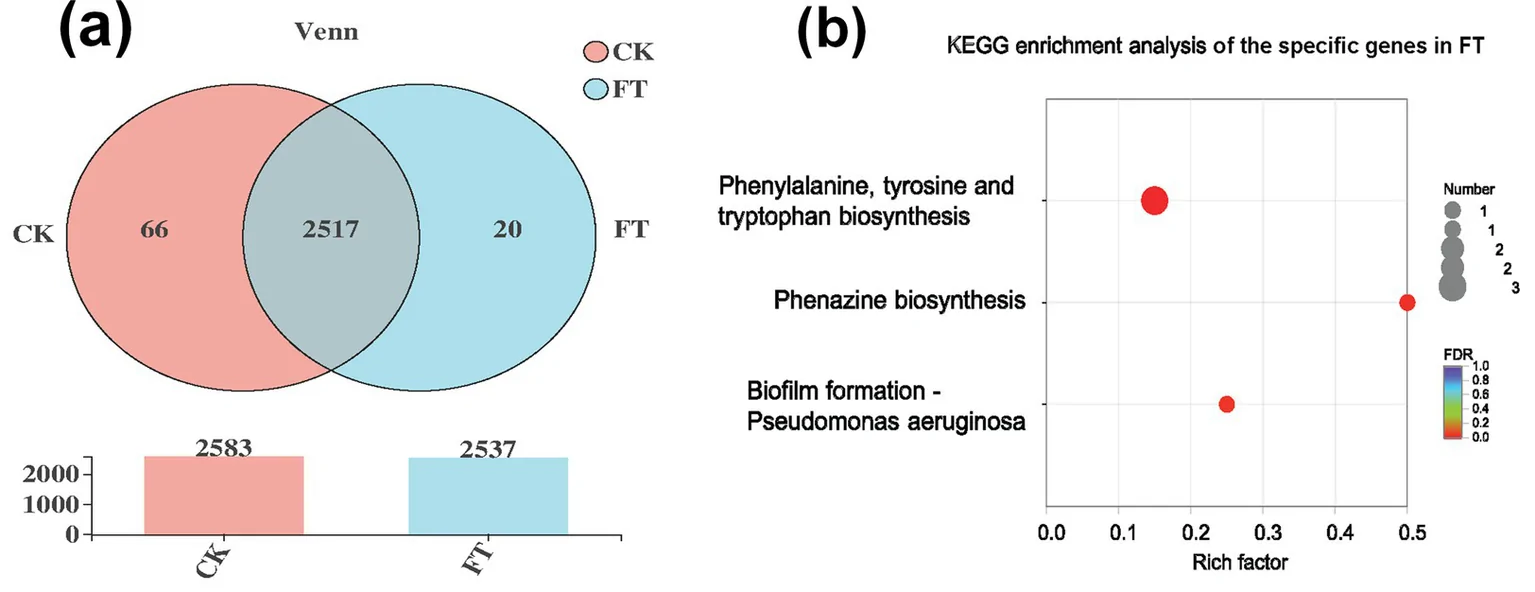
Transcriptome Venn diagram **(a)** and KEGG enrichment analysis of unique genes in FT **(b)**.

#### 3.3.2 Global remodeling of energy and material metabolism: coordinated regulation of ribosome, fatty acid synthesis, energy metabolism, and organic acid production

In response to freeze–thaw stress, QZW5 initiated a coordinated transcriptional reprogramming involving multiple core metabolic pathways. The central feature is the widespread suppression of energy-intensive biosynthetic processes coupled with fine-tuning of energy metabolism, collectively serving a survival-first strategy.

Freeze–thaw stress led to significant downregulation of a large number of genes in the ribosome biogenesis pathway (Supplementary Table 11). Ribosome assembly and protein translation are highly energy-consuming processes. Under stress conditions, downregulating the expression of these genes represents an “energy-saving strategy,” redirecting limited ATP resources from overall protein synthesis to critical processes essential for cell survival, such as membrane repair and antioxidant defense [14]. Consistent with this strategy, several other energy-intensive synthesis pathways were also broadly suppressed (Supplementary Table 9). In the fatty acid biosynthesis pathway, 12 key genes, including the initiation genes accA, accD and elongation/desaturation-related genes fabI and fabZ, were significantly downregulated. This not only directly reduces the consumption of ATP and reducing equivalents (Ding et al., 2025) but may also facilitate adaptive adjustments in membrane lipid composition to maintain membrane fluidity at low temperatures (Biscaia-Caleiras et al., 2026). The downregulation of key genes in the purine metabolism pathway (e.g., purD, purK) reflects a reduced demand for nucleic acid precursors under conditions where cell proliferation is inhibited, potentially redirecting metabolic flux toward the synthesis of products required for stress responses (Ragab, 2025). In the oxidative phosphorylation pathway, the expression of multiple F₀F₁ ATP synthase subunit genes (atpA, atpH, atpF, etc.) and electron transport chain-related genes (e.g., ndh) was downregulated (Supplementary Table 2). This is likely an adaptation to the compromised proton motive force caused by freeze–thaw damage, aiming to reduce “futile energy consumption” due to proton leakage and help minimize reactive oxygen species (ROS) generation from the electron transport chain (Chen et al., 2026; Shuai et al., 2025). Furthermore, the downregulation of the RNA degradosome polyphosphate kinase gene (ppk) may help preserve important mRNA templates under stress conditions.

While broadly suppressing energy-intensive processes, energy metabolism itself underwent specific adjustments. The expression of the key lactate metabolism gene DNA52_RS02900 (L-lactate dehydrogenase) was significantly downregulated, indicating a weakening of the primary lactate synthesis pathway. This could be due to cellular structural damage affecting substrate transport or gene expression, and may also help reduce intracellular osmotic pressure caused by lactate accumulation, thereby alleviating membrane damage (Tian et al., 2018). However, the expression of other lactate dehydrogenase genes remained stable, and the culture pH decreased significantly post-freeze–thaw, suggesting compensatory mechanisms maintained basic acid-producing capacity. In contrast, the expression of acetate kinase-related genes (DNA52_RS08290, DNA52_RS15280, DNA52_RS14845) showed no significant change before and after freeze–thaw. When the main lactate synthesis pathway was inhibited, the acetate kinase pathway, which converts acetyl-CoA to acetate with concomitant ATP generation, acted as an “energy stabilizer,” providing continuous energy supplementation for cell survival (Zhou et al., 2023). Stable acetate production also contributes to regulating the intracellular microenvironment, working synergistically with regulated lactate metabolism to maintain metabolic homeostasis (Xiao et al., 2025).

In summary, QZW5 implements an “energy-saving strategy” by globally downregulating energy-intensive pathways such as ribosome, fatty acid, and purine synthesis, as well as oxidative phosphorylation, thereby concentrating resources on survival-critical functions. Simultaneously, it performs differential fine-tuning of the lactate and acetate metabolism pathways (partially suppressing the former while maintaining the latter), ensuring basic energy supply and pH regulatory capacity while adapting to metabolic disruptions caused by stress. This multi-layered, coordinated metabolic reprogramming constitutes one of the core survival mechanisms of QZW5 in coping with freeze–thaw stress.

#### 3.3.3 Coordinated adjustment of metabolic cofactors and amino acid supply: stress responses in biotin, ribosome, and L-serine metabolism

To address the challenges in energy and material metabolism induced by freeze–thaw stress, QZW5 coordinately regulated a series of key metabolic pathways involving cofactor metabolism, translational machinery, and amino acid supply, collectively optimizing resource allocation for survival.

KEGG enrichment analysis revealed that both biotin metabolism and fatty acid biosynthesis pathways were significantly enriched among the downregulated differentially expressed genes (DEGs). Core enzyme genes, including fabI (enoyl-ACP reductase), fabZ (3-hydroxyacyl-ACP dehydratase), fabG (3-oxoacyl-ACP reductase), fabF (beta-ketoacyl-ACP synthase II), and the biotin–[acetyl-CoA-carboxylase] ligase gene birA, were consistently downregulated (Supplementary Table 13). Biotin is a key cofactor for acetyl-CoA carboxylase, the rate-limiting enzyme in fatty acid synthesis. Therefore, the downregulation of birA may not only limit the efficient utilization of biotin but also synergize with the downregulation of fab family genes to collectively inhibit fatty acid biosynthesis. This aligns with the global “energy-saving strategy,” as fatty acid synthesis is a highly energy-intensive process. Furthermore, reducing the synthesis of saturated fatty acids and potentially increasing the proportion of unsaturated fatty acids helps maintain cell membrane fluidity at low temperatures, consistent with cryo-electron microscopy observations of intact membrane structures (Ruiz et al., 2021). Concurrently, reduced biotin availability may indirectly affect other enzymatic reactions for which it serves as a cofactor, including the synthesis of certain antimicrobial substances, potentially relating to the observed changes in antibacterial activity (Guo et al., 2025).

Echoing the downregulation of metabolic pathways, a large number of genes in the ribosome pathway also showed significant changes (Supplementary Table 11). Ribosomes are the factories for protein synthesis, and their gene expression exhibited a complex regulatory pattern. The upregulation of some ribosomal protein genes may aim to enhance the translation efficiency of specific stress-responsive proteins (such as chaperones and antioxidant enzymes) to counteract freeze–thaw damage (Naik et al., 2025). However, the widespread downregulation of more ribosomal genes clearly indicates that, under energy-limited conditions, cells actively reduce the overall rate of protein synthesis to conserve energy, prioritizing resources for survival-critical processes like membrane repair. This “differential regulation” reflects a fine-tuned trade-off in translational capacity under stress.

As a precursor for protein synthesis, a donor of one-carbon units, and a precursor for various metabolites, the expression of L-serine-related genes also underwent adaptive adjustments. Most synthesis and metabolic genes showed a downregulation trend, such as the significant downregulation of homoserine dehydrogenase and serine acetyltransferase genes (Supplementary Table 14). This directly reduces the ATP and carbon unit consumption required for L-serine synthesis, representing the energy-saving strategy at the level of amino acid metabolism (Huang et al., 2025). Simultaneously, a few genes, such as the L-serine ammonia-lyase *α*-subunit gene (sdaAA) and a threonine/serine efflux protein gene (DNA52_RS12730), were upregulated. The former may maintain a minimal level of serine catabolism to supply energy or pyruvate, while the latter may help alleviate osmotic imbalance caused by freeze–thaw by regulating the intracellular/extracellular distribution of serine (Maharati et al., 2025). This pattern of “selective downregulation and upregulation” indicates that QZW5 does not simply shut down serine metabolism but reprograms it to ensure basic metabolic needs and intracellular homeostasis while conserving energy.

In summary, under freeze–thaw stress, QZW5 employs a coordinated transcriptional adjustment across different functional modules: coupling the downregulation of biotin and fatty acid synthesis pathways to conserve energy and modify membrane properties; differentially regulating ribosomal genes to balance translational demands and energy consumption; and reprogramming L-serine metabolic flux to balance synthetic costs with the maintenance of basic functions. These adjustments work synergistically to form a core adaptive strategy for optimizing metabolic resource allocation and prioritizing survival under resource-limited conditions.

#### 3.3.4 Upregulation of bacteriocin and drug resistance genes: enhanced defensive and detoxification capabilities under stress

To cope with the potentially intensified microbial competition and environmental toxicity pressure induced by freeze–thaw stress, QZW5 upregulated genes related to bacteriocin immunity and drug efflux (Supplementary Table 15). These genes include those encoding bacteriocin immunity proteins (e.g., DNA52_RS05350) and resistance-related genes mediating the efflux of tetracycline (DNA52_RS01475, encoding an MFS efflux pump) and anthracycline drugs (DNA52_RS06790, encoding a DrrA family ABC transporter ATP-binding protein).

Transcriptomic analysis reveals that the upregulation of these genes reflects an active defense strategy of QZW5 under stress. The enhanced expression of bacteriocin immunity protein genes helps the cells resist potentially excessive bacteriocins produced by themselves or surrounding flora in the imbalanced environment under stress, thereby maintaining a competitive advantage in microbial competition (Qiao et al., 2022). Concurrently, the upregulation of drug efflux pump genes directly improves the cell’s ability to actively expel antibiotics (e.g., tetracycline) or potential toxic substances (e.g., damage-related metabolites). This enhanced detoxification function helps maintain the fragile intracellular homeostasis under stress conditions, providing a relatively stable environment for critical repair processes (Zhang et al., 2015). This finding provides a molecular-level explanation for the previously observed phenotype of enhanced antibiotic resistance in the strain after freeze–thaw.

In summary, the upregulation of bacteriocin immunity and drug efflux genes is part of the multi-layered defense system constructed by QZW5 under freeze–thaw stress. Although not a direct freeze-damage repair mechanism, it indirectly supports cell survival in the stressful environment and ensures the progression of core repair processes by enhancing resistance to biotic competitive pressure and chemical toxicity.

### 3.4 Proteomic and metabolomic analysis: revealing coordinated protein and metabolite responses under stress

Proteomic analysis indicated that freeze–thaw stress induced extensive reprogramming of protein expression in QZW5. Volcano plot analysis of differentially expressed proteins (DEPs) revealed significant changes in the expression of proteins related to L-serine, biotin amide, quinones, and aniline (Figure 4a). KEGG pathway enrichment analysis further showed that DEPs were significantly enriched in pathways such as purine metabolism, ABC transporters, vancomycin resistance, pyrimidine metabolism, and oxidative phosphorylation (Figure 4b). Among these, downregulated DEPs were primarily enriched in pathways like oxidative phosphorylation (Figure 4c), while upregulated DEPs were enriched in pathways including purine metabolism, ABC transporters, and pyrimidine metabolism (Figure 4d). These changes correspond to perturbations at the metabolite level, together outlining a coordinated adaptive strategy of the cell in response to stress.

**Figure 4:**
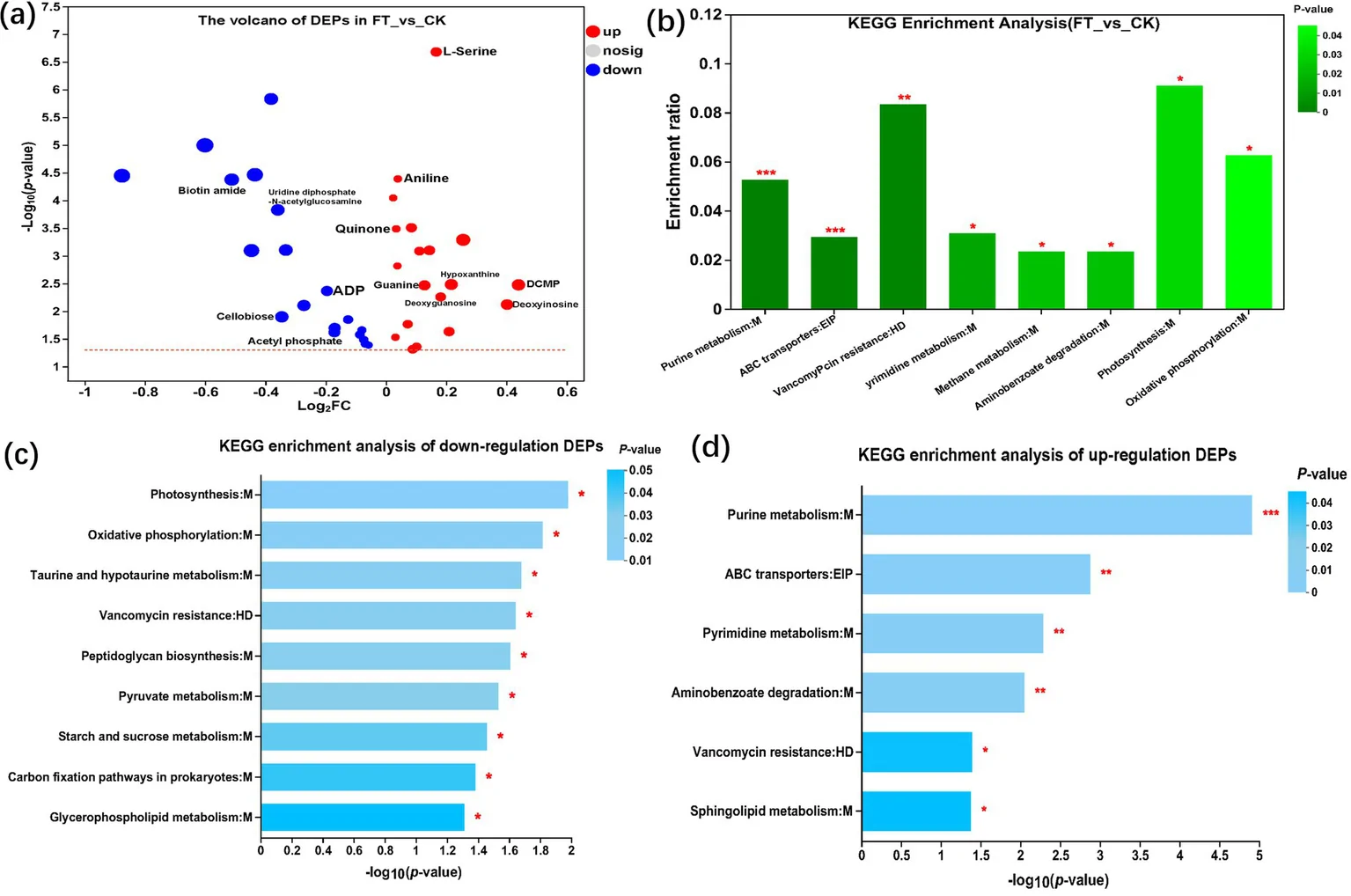
Volcano plot of differentially expressed proteins (DEPs) between CK and FT groups **(a)**, and KEGG metabolic pathway enrichment of DEPs **(b, c, d)**.

The downregulation of proteins related to oxidative phosphorylation (Figure 4c) aligns with the downregulation of F₀F₁ ATP synthase subunit genes observed in the transcriptome, jointly reflecting an “energy-saving strategy” under conditions of membrane damage and impaired proton motive force. Concurrently, the upregulation of genes and proteins related to ABC transporters (Figure 4d) significantly enhances the cell’s material transport capacity. This helps improve the efficiency of nutrient uptake and harmful substance efflux in the stressful environment, working synergistically to maintain intracellular homeostasis (Feng et al., 2025).

Although genes related to purine metabolism were downregulated at the transcriptional level, the proteome showed upregulation in this pathway (Figure 4d), and metabolomic analysis revealed the accumulation of purine metabolites such as hypoxanthine and guanine. This “gene-protein-metabolite” differential regulatory pattern (Carrey et al., 2013) may indicate that while the cell slows down synthesis, it accumulates or recycles nucleotide precursors through other pathways, preparing raw materials for potential nucleic acid repair after freeze–thaw damage.

Regarding amino acid metabolism, the upregulation of L-serine and its related proteins provides raw material support for the synthesis of stress-related proteins. Simultaneously, the downregulation of some amino acid derivatives may reduce the synthesis of non-essential proteins to conserve energy, consistent with the “survival-first” metabolic principle (Feng et al., 2025; Liu et al., 2021). Specific changes were also observed in membrane lipid metabolites. For instance, the upregulation of heneicosanoic acid and ceramide may help enhance membrane stability, while the downregulation of certain glycerophospholipids may optimize lipid composition to maintain membrane fluidity. These changes correspond with the cryo-electron microscopy observation of damaged yet functional membrane structures and the downregulation of fatty acid synthesis genes in the transcriptome. Together, they form a multi-layered, coordinated defense system spanning gene expression, metabolite composition, and membrane structure and function (Liu et al., 2025).

### 3.5 Integrated genomic and transcriptomic analysis

Integrated analysis of genomic, transcriptomic, and metabolomic data indicates that QZW5 systematically adapts to freeze–thaw stress through coordinated changes in genes and metabolites across several core pathways. Nine co-enriched pathways were identified, with ABC transporters, purine metabolism, and the ribosome pathway being the most prominent (Figure 5).

**Figure 5:**
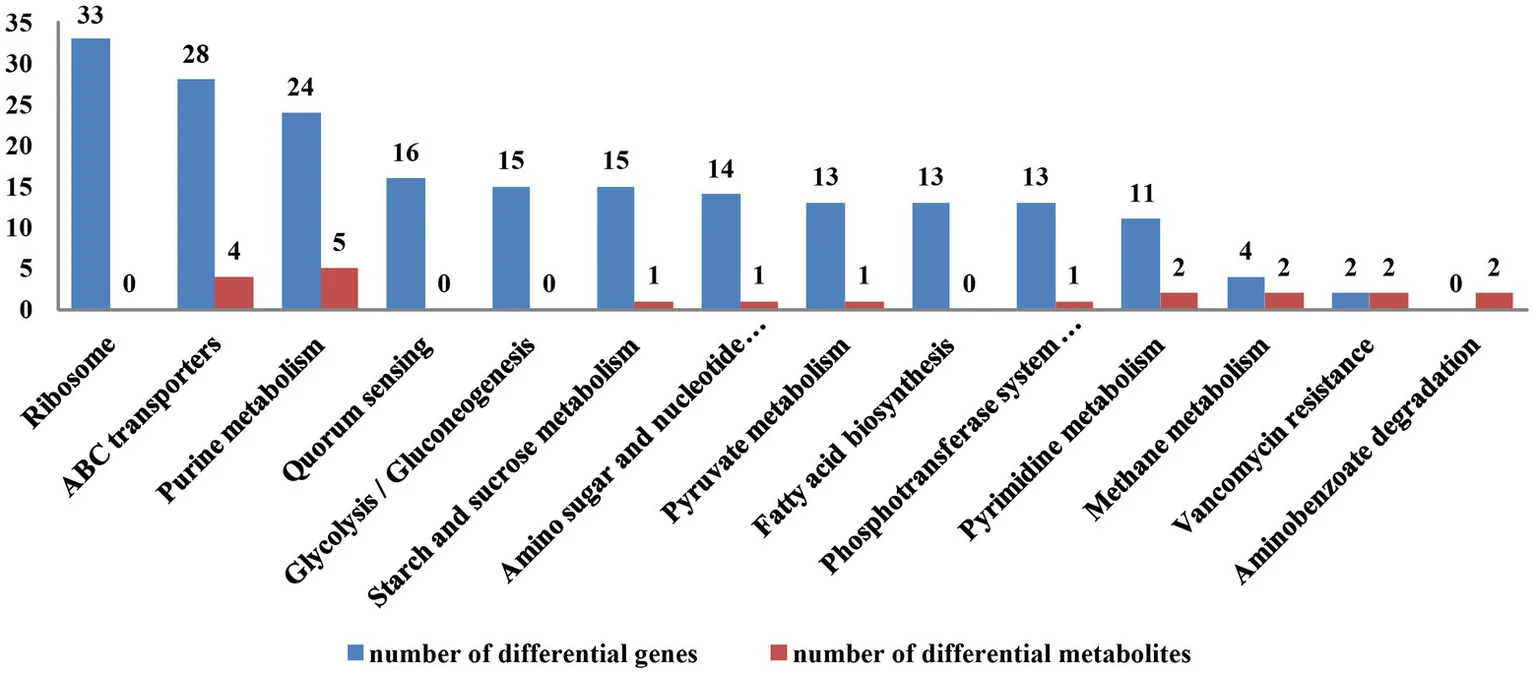
The top 10 KEGG pathways containing the largest number of differentially expressed genes/differentially expressed metabolites.

The ABC transporters pathway contains the highest number of differential molecules (28 DEGs, 4 metabolites), serving as a hub for regulating material homeostasis. The upregulation of its genes enhances nutrient uptake and toxin efflux capacity, while changes in metabolites (e.g., upregulation of L-serine and deoxynucleosides, downregulation of cellobiose) reflect an optimization of raw material supply under stress. The activation of the purine metabolism pathway (24 DEGs, 5 metabolites) addresses the dual needs of “repair and energy supply.” The accumulation of metabolites such as hypoxanthine and guanine provides precursors for nucleic acid repair, while coordinated gene expression adjusts nucleotide levels to ensure basic energy supply, explaining the mechanism by which the strain maintains basal metabolism after stress (Zhang et al., 2025).

The ribosome pathway, although lacking significantly changed metabolites, exhibits differential expression of 33 DEGs (some upregulated, others downregulated), reflecting a fine-tuned trade-off in protein synthesis. This aims to meet the demand for repair protein synthesis while achieving energy conservation. The oxidative phosphorylation pathway displays a typical “gene-metabolite” interactive regulatory pattern. The upregulation of specific complex subunit genes supports ATP synthesis to drive repair, while the downregulation of other genes reduces futile energy consumption caused by membrane damage. Combined with changes in metabolites such as coenzymes, this forms an elastic regulatory network for energy metabolism (Enriquez, 2016), consistent with the previously observed downregulation of ATP synthase genes and decreased acetyl phosphate levels.

Furthermore, the downregulation of uridine diphosphate-N-acetylglucosamine corresponds to transcriptional changes in cell wall synthesis-related genes (e.g., ddl and murG). This suggests that under stress, the cell may coordinately reduce the synthesis of cell wall precursors, instead optimizing existing peptidoglycan metabolism to enhance cell wall toughness, exemplifying the synergy between structural repair and metabolic adaptation (Xu et al., 2024).

### 3.6 Validation of differentially expressed genes by quantitative real-time PCR

The qPCR results (Table 2) validated the expression changes of several key genes. The significant downregulation of fatty acid synthesis gene fabZ, purine synthesis gene purN, lactate dehydrogenase gene ldh, and F₀F₁ ATP synthase subunit genes aligns with the core stress adaptation strategies of “inhibited fatty acid synthesis” and “contracted energy metabolism” revealed by transcriptomic and metabolomic analyses, and also corroborates the membrane damage phenotype observed via cryo-electron microscopy. Conversely, the upregulated genes, including the bile salt hydrolase gene DNA52_RS12630 and NAD(P)/FAD oxidoreductase genes, may participate in membrane lipid remodeling or osmoregulation and enhance ROS scavenging capacity, respectively. These work synergistically with the “upregulated glutathione metabolism” to mitigate oxidative damage (Wang et al., 2026). These results validate the core regulatory mechanisms employed by QZW5 in response to freeze–thaw stress at the molecular level.

**Table 2:** Results of quantitative real-time PCR.

Gene_id	Gene name	Gene description	Significant	Regulate	CK	FT
DNA52_RS05455	fabZ	3-hydroxyacyl-ACP dehydratase FabZ	Yes	Down	2070.66	61.99
DNA52_RS09925	purN	Phosphoribosylglycinamide formyltransferase	Yes	Down	589.40	26.10
DNA52_RS05460	DNA52_RS05460	ketoacyl-ACP synthase III	Yes	Down	904.52	29.84
DNA52_RS13990	DNA52_RS13990	30S ribosomal protein S6	Yes	Down	1967.73	43.91
DNA52_RS08850	DNA52_RS08850	F0F1 ATP synthase subunit alpha	Yes	Down	155.17	8.65
DNA52_RS08855	DNA52_RS08855	F0F1 ATP synthase subunit delta	Yes	Down	226.16	21.60
DNA52_RS02770	DNA52_RS02770	NAD(P)/FAD-dependent oxidoreductase	Yes	Down	124.96	16.53
DNA52_RS02900	DNA52_RS02900	L-lactate dehydrogenase	Yes	Down	42.76	7.29
DNA52_RS12630	DNA52_RS12630	Choloylglycine hydrolase family protein	Yes	Up	83.97	778.42

In recent years, research on probiotic functionality has gradually expanded beyond the application of live viable strains, driving the emerging concepts of paraprobiotics and postbiotics. Paraprobiotics refer to non-viable inactivated microbial cells or structurally intact dead bacterial biomass, while postbiotics are defined as soluble bioactive metabolites, enzymes, cell-wall fragments and small-molecule compounds secreted by probiotic strains after fermentation or cell lysis. Compared with conventional live probiotics that are susceptible to viability loss induced by freeze–thaw damage, temperature fluctuation and long-term storage, both paraprobiotics and postbiotics retain stable physiological bioactivities independent of bacterial viability. Notably, previous research confirmed that paraprobiotics can continuously maintain their regulatory efficacy against diet-triggered intestinal dysbiosis throughout the entire product shelf life without obvious activity attenuation (Yolmeh et al., 2026). In the present study, Lactiplantibacillus plantarum QZW5 survived repeated freeze–thaw cycles with partial ultrastructural injury but stable biofilm formation capacity and retained abundant intracellular functional metabolites. Therefore, QZW5 possesses promising developmental potential as a raw material for paraprobiotic and postbiotic products; its derived bioactive components can exert persistent health benefits during long-term cold storage, which greatly improves the application stability of this strain in frozen-food-related commercial formulas.

## 4 Conclusion

This study systematically elucidated the survival mechanisms of *Lactiplantibacillus plantarum* QZW5 under repeated freeze–thaw stress by integrating biochemical analysis, environmental cryo-electron microscopy, and multi-omics technologies. After 10 cycles of alternating freezing at −20 °C and thawing at 20 °C, QZW5 maintained a high survival rate of 85.20%, while exhibiting a gradual decrease in pH, reduced antibacterial activity, enhanced antibiotic resistance, and stable biofilm-forming capacity. Cryo-electron microscopy confirmed structural damage, including serrated cell edges and intracellular granules. Omics analyses revealed that QZW5 enhances stress adaptation by activating pathways such as glyoxylate and dicarboxylate metabolism, ABC transporters, and purine/pyrimidine metabolism. Concurrently, the upregulation of bacteriocin immunity and drug resistance genes strengthens its defensive functions. In contrast, pathways including oxidative phosphorylation, fatty acid synthesis, and ribosome metabolism were broadly downregulated. Notably, the significant downregulation of the key L-lactate dehydrogenase gene (DNA52_RS02900) indicates an energy conservation strategy for survival. The specific activation of genes involved in phenylalanine/tyrosine/tryptophan biosynthesis, phenazine production, and biofilm formation collectively establishes a protective network that enhances freeze–thaw tolerance. This study provides both a theoretical foundation and technical support for the application of QZW5 as a functional starter culture in frozen foods. Future research could focus on functional validation of key differential genes and explore pretreatment strategies, such as cold acclimation and exogenous protectants, to further improve the freeze–thaw tolerance of the strain and promote its practical application in the frozen food industry.

## Data Availability

The sequencing dataset has been deposited in the NCBI Sequence Read Archive (SRA) under BioSample accessions SAMN62923436–SAMN62923446. The metabolomics dataset has been deposited in the National Genomics Data Center (NGDC) OMIX database under BioProject accession PRJCA073552, available under accession numbers OMIX020292-01 through OMIX020292-55.
